# Poly(phosphazene)-Coatings for Stabilizing Silicon Thin-Film Anodes in Lithium-Ion-Batteries

**DOI:** 10.1021/acsami.6c04115

**Published:** 2026-06-01

**Authors:** Nis-Julian H. Kneusels, Ben E. Smith, Kieran Mylrea, Yanting Jin, Zachary Ruff, Robert S. Weatherup, Dominic S. Wright, Clare P. Grey

**Affiliations:** The Yusuf Hamied Department of Chemistry, 150385 University of Cambridge, Lensfield Road, Cambridge CB2 1EW, U.K.

**Keywords:** silicon anode, solid electrolyte interphase, poly(phosphazenes), thin-film electrodes, data analysis

## Abstract

Silicon is a promising anode material for lithium-ion batteries, but its practical use is limited by several factors: first, upon lithiation, silicon forms highly reduced phases, which themselves reduce the electrolyte unless the surfaces are passivated; second, silicon undergoes large volume changes during charge and discharge. Both processes lead to capacity loss, and their effects need to be mitigated to improve cell lifetime. To address these fundamental roadblocks, in this work, flexible poly­(phosphazenes) are explored as Si coatings. Amorphous silicon thin-film electrodes are used to simplify the coating procedure and explore the role of these coatings in limiting electrolyte reduction. Two short-chain linear poly­(phosphazenes) were prepared and cross-linked on the surfaces of the electrodes. The electrochemical performance of the electrodes and the accompanying structural changes of the electrode material were investigated. A partially fluorinated phosphazene coating was shown to improve the cycle life of the silicon thin-film anode most, allowing more than 500 cycles to be achieved with improved Coulombic efficiencies compared to the other electrodes. For analytical purposes, the Navani Python package was developed to track lithiation potentials from incremental capacity analysis during cycling. The package was used to measure how the polymer coatings affected lithiation potentials of the silicon electrode, giving information about the changes in mechanical stress during cell aging, and changes in overpotential due to the buildup of the solid electrolyte interface (SEI).

## Graphical abstract

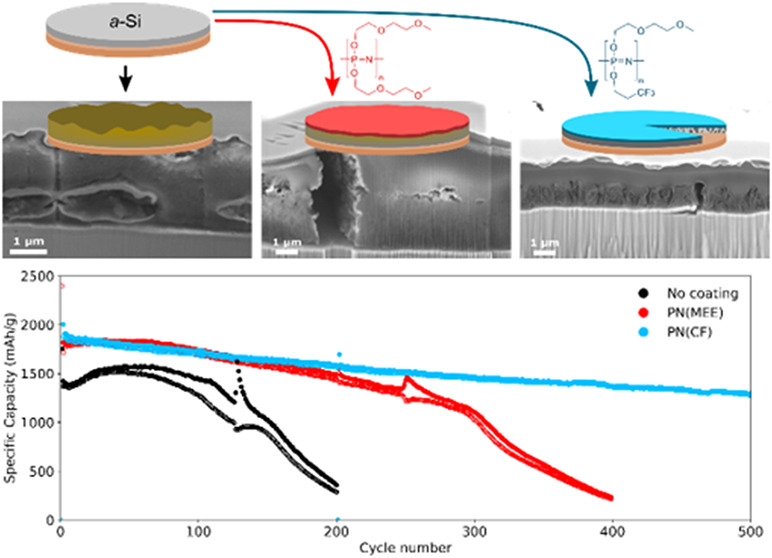

## 1 Introduction

Lithium-ion batteries (LIBs) are crucial to the development and employment of sustainable e-mobility, consumer electronics, and grid-scale energy storage solutions. Currently, several high-capacity electrode materials containing environmentally sustainable elements are being explored, with the aim of increasing energy density. Silicon stands out as a next-generation anode material due to its high gravimetric capacity (3579 mAh g^–1^ for Li_15_Si_4_ compared to 372 mAh g^–1^ for LiC_6_) and low operating potential (<0.4 V vs Li/Li^+^), while also being relatively cheap.^1^ However, a major roadblock to its broad commercial application in LIBs is the large volume expansion during lithiation (>300%), leading to continuous cracking and ultimately pulverization of the material.^2^ This is accompanied by additional growth of the solid electrolyte interphase (SEI) that forms when the organic electrolyte decomposes on the anode’s highly reactive surface. The continuous formation of SEI, particularly when the silicon anode is lithiated (i.e., at high states of charge in a full cell), removes active lithium from the cell and leads to increased cell impedance and capacity fade. Cracking and pulverization of the silicon leads to electronically “dead” silicon that is no longer connected to the current collector; this dead material will not participate in future charge–discharge cycles and may trap lithium that is no longer accessible, ultimately resulting in capacity loss of the cell. As a result of these challenges, commercial use of Si has been largely limited to its application as a minor component in Si-graphite composites or to applications where high capacity and relatively few charge–discharge cycles are required. Multiple strategies have been presented to mitigate the capacity loss, including the use of micro- and nanosized silicon materials (e.g., nanoparticles,^3^ nanowires,^3–5^ thin films^6,7^ ), electrolyte additives,^8^ and applying passivating and shape-preserving artificial SEIs,^9,10^ all of which have shown a positive effect, to varying degrees, on the long-term cycling capabilities.

Among the nanomaterials, silicon thin films present versatile, controllable model anodes for studying cycling conditions and testing the performance of electrolyte additives and surface coatings. Thin films can be manufactured with controlled thickness and density from physical or chemical vapor deposition directly onto a current collector, which ensures initial electrical contact, and allows electrochemical cycling in half or full cells without adding conductive carbon additives and binders. This allows the effects of surfaces/additives to be explored, at least in the first cycles, without having to consider the effects of poor electrical wiring (and thus dead silicon). However, silicon thin films have some drawbacks, their two-dimensional nature resulting in large compressive and tensile stresses during lithiation and delithiation, leading to fracturing and delamination of the silicon layer from the current collector, particularly during prolonged cycling. The structural changes of silicon have been investigated, and lithiation pathways have been proposed where continuous lithiation and delithiation leads to cracks and the formation of island-like structures of the silicon material.^11–13^ Stress-potential coupling has also been observed in silicon materials during electrochemical cycling, which affects the anode materials’ thermodynamics, resulting in changes in voltage observed during cycling.^14,15^ This change in potential can be tracked by observing the potential of the two main voltage plateaus during discharge. Typically, flat voltage plateaus arise during a two-phase reaction between well-defined crystalline phases. In silicon, however, these “plateaus” are sloping and correspond to the transition of the amorphous (*a*-) silicon phase, *a*-Si, to another amorphous phase with approximate composition *a*-Li_2_Si, and then from *a*-Li_2_Si to *a*-Li_3.5_Si and higher lithium contents at ∼0.25 and 0.09 V vs Li/Li^+^, respectively.^16^ Here, the term “phase” is used because the processes are associated with characteristic lithiation voltages implying distinct amorphous structures, as discussed in previous work.^16^ Further lithiation of *a*-Li_3.5_Si (≤50 mV) can lead to the formation of the crystalline Li_3.75_Si-phase, which leads to accelerated degradation.^12^

Therefore, cycling conditions play an important role in controlling the degree of lithiation and, hence, the degradation of the silicon electrode. Reducing the capacity by increasing the lower cutoff voltage limits the lithiation and thus volume expansion of the silicon, reducing the stress on the material, allowing longer cycling as a trade-off. During cycling, the potentials of the plateaus associated with the different lithiation processes can be tracked by measuring the potential of the corresponding peak in the differential capacity plot (dQ/dV). Once cracking has occurred, tensile and compressive stresses during cycling are reduced, as the silicon islands can expand more freely. This stress relief leads to an increase in the lithiation potential of the two main discharge plateaus.^14,15^ In contrast, the buildup of SEI and subsequent increase in impedance lead to a decrease in the measured potential of the voltage plateaus when cycling using typical currents.

The use of electrode coatings may also help to limit the degradation. Examples include carbon interlayers^17^ and ion conductors such as lithium phosphorus oxynitride^18,19^ to stabilize silicon thin-film anodes and shape-preserving shells for silicon nanoparticles.^9,10^ Coatings applied by either physical or chemical vapor deposition (PVD or CVD) are more costly to scale up, while coatings formed by calcination of a precursor are less flexible. Solution-based approaches provide a gentler and more easily controlled way of introducing artificial coatings. There are, however, only a few examples of such soft coatings. These include molecular grafts with short- and long-chain molecules to successfully increase the cycle life of silicon nanoparticle anodes.^20,21^ Polymer-based coatings could also provide more versatility where physical deposition methods are difficult to use.

From the vast number of polymers in existence, poly­(phosphazenes), a class of organic–inorganic hybrid polymers, stands out as promising candidates. They are based on thermodynamically stable P–N bonded backbones, −(R_2_P = N)_n_–, and have been tested as liquid and gel electrolytes due to their tunable structural and chemical properties.^22–26^ Their chemical inertness and highly varied mechanical characteristics (such as flexibility) have led to applications in biomedicine and engineering some decades ago.^27,28^ Tuning of the chemical and mechanical properties is readily achieved by altering the substituents (R) on the P­(V)-atoms of the P–N-backbone. Previously, the introduction of polyether groups onto the poly­(phosphazene) chain, having multiple coordination sites for cations, has been used to introduce greatly increased lithium conductivity in the phosphazene material.^22^ Examples by Wiemhöfer et al., using the poly­(phosphazenes) as in-gel electrolytes for lithium metal anodes, have shown that they can stabilize the lithium metal surface, suppressing dendrite formation and allowing long-term cycling.^25,26,29–33^ The brush-like structures of the poly­(phosphazenes), combined with the nonlinear arrangement of their backbones, lead to highly elastic polymers. Furthermore, poly­(phosphazenes) act as flame-retardants which additionally increases battery safety.^34–37^

The inherent elasticity of poly­(phosphazenes) and their inert nature under harsh reducing conditions make them promising candidates as artificial SEIs for silicon anodes, showing potential to accommodate volumetric expansion and protect the silicon surface. Herein, we study the structural evolution of poly­(phosphazene)-coated silicon thin-film anodes (illustrated in Figure 1) during electrochemical cycling. Employing the living-cationic polymerization,^38,39^ soluble, short-chain polymers were prepared and functionalized with two different side groups. By comparing two poly­(phosphazenes), we explore the impact of the organic substituents on the polymer and the electrode performance. We show that with all-polyether-substituted side groups, to form the polymer referred to as **PN­(MEE)**, a higher degree of cross-linking is obtained, which provides stability against early delamination. Partial substitution with trifluoroethoxide groups (polymer **PN­(CF)**) stabilizes the silicon nanostructure that forms during cycling from the silicon thin-film anode even better. This coating extends the cycle life by limiting SEI formation, yielding a high Coulombic efficiency (CE) of >99% in a lithium half-cell over more than 500 cycles. The work demonstrates that poly­(phosphazene)-based SEIs provide a potent strategy to limit the degradation of silicon anodes in LIBs, while the work also highlights the challenges associated with continual SEI formation.

**Figure 1:**
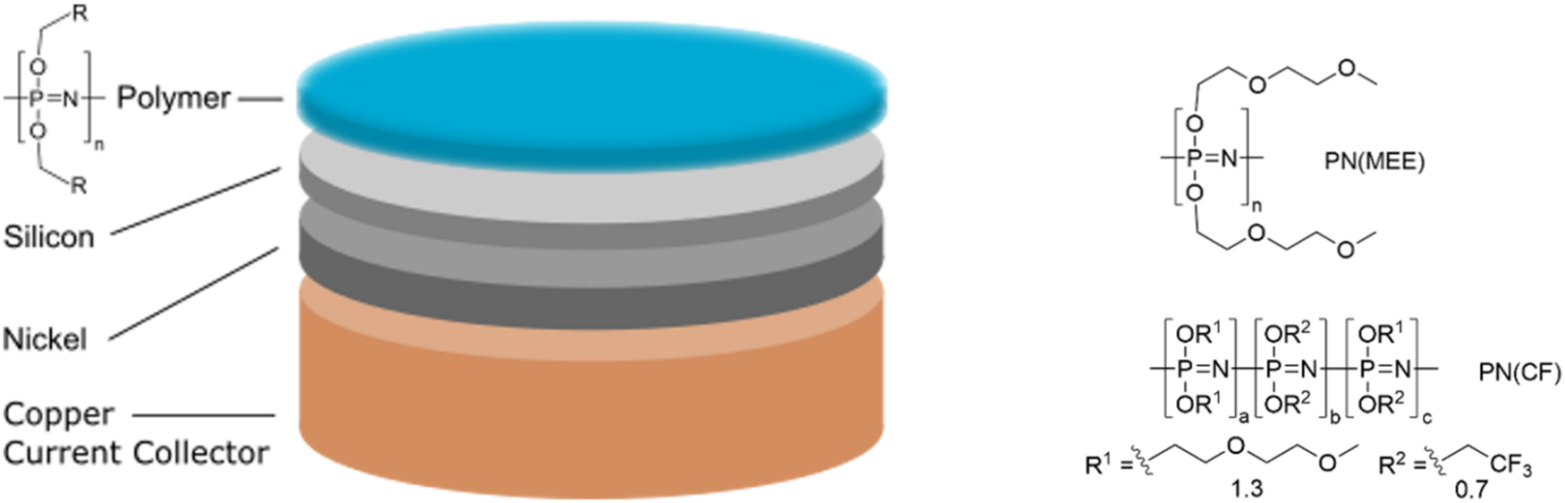
Schematic of the polymer-coated silicon thin-film anodes used in this study comprising of a copper current collector, a 250 nm nickel interlayer, a 100 nm silicon thin-film, and a poly­(phosphazene) coating and the structures of **PN­(MEE)** and **PN­(CF)**.

A significant challenge in battery research is the fragmented nature of battery cycling and electrochemical data storage: although all battery cyclers measure and store the same data—current, time, and voltage—different commercially available cyclers use proprietary software and different data formats to store cycling data. Additionally, the level of analysis offered by the software varies. For example, Biologic includes dQ/dV analysis in their software, while at the time of writing Landt, and many other manufacturers, do not. Since the cells cycled in this paper used a Landt cycler, an alternative method was needed to measure the dQ/dV and track the stress-potential coupling, and overpotential of the cell. To address this issue, the Navani Python package was developed and used. This package can read electrochemical data files from Biologic, Landt, Neware, Ivium, and Arbin battery cyclers, exporting them in a standardized, easy-to-read format. This standardization facilitates consistent analysis across different battery cycler manufacturers. The package also includes functions to calculate relevant measurements from the standardized data, e.g., capacity, Coulombic efficiency (CE), and the average voltage difference between charge and discharge for each cycle. Furthermore, Navani includes functions for calculating the dQ/dV and dV/dQ for each cycle, and tools to fit dV/dQ half-cell profiles to the full-cell profile using the approach outlined by Bloom et al.,^40^ extracting information about different degradation modes. The development of Navani enabled the tracking of the dQ/dV peak potentials over hundreds of cycles, and therefore information about the stress-potential coupling in the silicon anode.

## 2 Experimental Section

### 2.1 Materials

Phosphorus trichloride and sulfuryl chloride were obtained from Sigma-Aldrich and distilled under nitrogen atmosphere prior to use. 2-(2-methoxyethoxy) ethanol (99%), hexamethyldisilazane (99%), and sodium hydride (dry powder, 90%) were obtained from Sigma-Aldrich, and trifluoroethanol (99.8%, ultrapure) was obtained from Acros Organics and used as received. *n-*Hexane, diethyl ether, tetrahydrofuran, and chloroform-*d* were dried over a suitable drying reagent and distilled before use.^41^ Cold-rolled copper foil (0.018 mm) was used as a current collector. Electrolyte solution (LP30:1m LiPF_6_ in 1:1 ethylene carbonate and dimethyl carbonate - battery grade) was obtained from Sigma-Aldrich and used as received.

### 2.2 Polymer Synthesis

#### 2.2.1 Synthesis of Trichloro­(trimethylsilyl)­phosphoranimine

The monomeric phosphazene precursor was synthesized following the improved procedure described by Wang et al.^38^ A total volume of 200 mL *n*-butyl lithium solution (0.8 m in hexanes, 160 mmol, 1.0 equiv) was slowly added to an ice-chilled solution of hexamethyldisilazane (33.5 mL, 160 mmol, 1.0 equiv) in 50 mL *n*-hexane (abs.) over the course of 2 h. The reaction mixture was slowly warmed to room temperature and left to stir for 18 h. All volatiles were removed under reduced pressure to yield crystalline lithium hexamethyldisilazide. The solid was redissolved in 300 mL diethyl ether (abs.) and cooled to 0 °C. Phosphorus trichloride (14 mL, 160 mmol, 1.0 equiv) was added slowly over 30 min. The mixture was warmed to room temperature and stirred for another 2 h. The mixture was then cooled to 0 °C and sulfuryl dichloride (12.9 mL, 160 mmol, 1.0 equiv) was added slowly via syringe over 30 min. After stirring for another 2 h at room temperature, all solids were filtered off over Celite and volatiles were removed under reduced pressure, keeping the temperature at 0 °C. The liquid product subsequently was distilled (23 °C, 1.3 × 10^–1^ mbar) to yield colorless trichloro­(trimethylsilyl)-phosphoranimine (24.0 g, 92 mmol, 76%). ^31^P­{^1^H} NMR (162.0 MHz, Et_2_O): δ = −58.8 ppm (Lit. δ = −53.0 ppm in CDCl_3_).^38^

#### 2.2.2 Synthesis of the PN­(MEE) and PN­(CF)

Trichloro­(trimethylsilyl)­phosphoranimine (2.3 g, 1.0 equiv, 10.2 mmol) was added to a solution of phosphorus pentachloride (12 mg, 0.01 equiv, 57.6 μmol) in 45 mL toluene (abs.). The solution turned opaque after an hour and was left to stir for 2 days until it cleared. At this stage, the ^31^P­{^1^H} NMR spectrum showed complete conversion. The solvent was removed, and the crude product was taken up in THF (abs.) and slowly added to a solution of freshly prepared sodium alkoxide from sodium hydride (from a glovebox) and the appropriate alcohol at 0 °C: 2.2 equiv sodium methoxy­(ethoxy)­ethoxide in THF (abs.) and stirring for 5 days to form **PN­(MEE)**; 1.2 equiv methoxy­(ethoxy)­ethoxide in THF (abs.) followed by excess sodium trifluoroethoxide in THF (abs.) to form **PN­(CF)**. The solvent was removed under reduced pressure, and the residue was dissolved in deionized water. The product was purified by dialysis using cellulose tubing with a molecular weight cutoff of 12–14 kDa over 5 days in deionized water, changing the water multiple times each day. Removing the water, the polymers were obtained as highly viscous yellow liquids. **PN­(MEE)**: 2.2 g, 76%. ^31^P­{^1^H} NMR (162.0 MHz, THF-*d*_8_): δ = −8.01 ppm. ^1^H NMR (399.6 MHz, THF-*d*_8_): δ = 4.11 (bm, 2 H), 3.67 (bm, 2 H), 3.62 (bm, 2 H), 3.48 (bm, 2 H), 3.30 (s, 3 H) ppm. ^13^C­{^1^H} NMR (100.5 MHz, THF-*d*_8_): δ = 72.0, 70.4, 70.2, 65.0, 58.0 (CH_3_) ppm. **PN­(CF)**: 2.4 g, 80%.^31^P­{^1^H} NMR (162.0 MHz, THF-*d*_8_): δ = −6.74 (b, 25%), −7.7 to −9.0 (b, 75%) ppm.^1^H NMR (399.6 MHz, THF-*d*_8_): δ = 4.45 (b, 2 CH_2_OCF_3_), 4.14 (b, 2 H), 3.65 (b, 2 H), 3.59 (b, 2 H), 3.46 (bm, 2 H), 3.28 (s, 3 H) ppm.^13^C­{^1^H} NMR (100.5 MHz, THF-*d*_8_): δ = 71.9, 70.2, 70.0 (b), 65.5 (b), 62.3 (q, CH_2_OCF_3_, ^3^JC-F = 34 Hz), 57.9 (CH_3_) ppm. ^19^F NMR (376.0 MHz, THF-*d*_8_): δ = −76.0 (m) ppm.

### 2.3 Gel-Permeation Chromatography (GPC)

Molecular weights (*M_n_*, *M*_w_) and dispersity values (Đ) of the synthesized polymers were determined by size exclusion chromatography (SEC) with dimethylformamide (DMF, HPLC grade, VWR) as eluent containing 1 g L^–1^ lithium bromide (98%, Sigma-Aldrich) using a Shimadzu LC system equipped with a diode-array detector (SPD-M20A DAD) and a refractive index detector (RI, Viscotek VE3580). One precolumn (50 × 8 mm), followed by three PSS GRAM gel columns (300 × 8 mm), were applied with a flow rate of 1.0 mL min^–1^ at 25 °C. The diameter of the gel particles measured 10 μm, the nominal pore widths were 100 Å once and 3000 Å twice, respectively. Samples were dissolved in DMF and filtered prior to analysis. The method was calibrated to PSS standard solutions.

### 2.4 Mass Spectrometry (MALDI TOF)

Matrix-assisted laser desorption/ionization time-of-flight (MALDI TOF) mass spectrometry was used to characterize the poly­(phosphazenes). Spectra were recorded on a Bruker ultrafleXtreme MALDI instrument with a TOF detector by the University of Cambridge Department of Chemistry Mass Spectrometry section.

### 2.5 Thermogravimetric Analysis (TGA)

Thermogravimetric analysis (TGA) of samples was performed on a Mettler Toledo TGA/DSC 2 STARe System. Samples of 30–50 mg were heated to 800 °C at a rate of 10 °C/min. Measurements on samples were performed under a constant flow of N_2_ (100 mL/min).

The initial decomposition temperature was determined using the extrapolated onset method, in which the steepest point of descent is detected, a tangent is extrapolated back to 100% of mass remaining, and the intersect temperature is taken as the decomposition temperature. The initial mass loss observed before this temperature is assigned to solvent and water loss, as samples were stored in air.

### 2.6 Silicon Thin-Film Preparation

12 mm disks of cold-rolled copper foil were used as substrates. Si thin-film anodes (∼100 nm) were prepared in two steps. First, the disks were coated with 250 nm nickel using a custom DC sputter coater (base pressure ≤ 2.0 × 10^–5^ mbar) from a pure nickel target (PI-KEM, <99.999%), which was followed by approximately. This was followed by 100 nm of silicon using a RF sputter coater (CCR Technology, base pressure ≤ 1.0 × 10^–5^ mbar) from an undoped silicon wafer target (PI-KEM, <99.999%). The total amount of silicon deposited varied slightly depending on the distance of the individual disks from the center of the target leading to variations in the mass of Silicon deposited in the different films. Thicknesses of the deposited films were determined by profilometry using pieces of masked silicon wafer as observers. The mass of active material is estimated based on geometrical parameters, assuming a density of 2.33 gcm^–3^ for the amorphous silicon which yields ∼28 μg. However, weighing various anodes the masses to vary between 22 and 27 μg. Given that the weight of the copper substrate (∼21 mg) holds the substantial fraction of the weight, and regarding the multistep preparation process, these measurements are relatively unreliable. Therefore, weights of 28 μg are assumed for the following capacity calculations which will lead to discrepancies in the absolute capacities when comparing samples of up to 25%.

### 2.7 Spin Coating

Solutions were prepared with 10 wt % of the respective polymer and 2 wt % of benzophenone as an UV cross-linking agent in toluene and sonicated until clear. Polymer solutions (0.1 mL) were drop-cast onto electrode disks mounted on a Delta 10 TT spin-coater (SUESS MicroTec Lithography GmbH). The disks were spun at 300 rpm for 30 s, followed by 2500 rpm for 120 s and left to dry for a few hours before cross-linking using a UV lamp (low pressure Hg, 450 W) for 10 min. The electrodes were dried in a vacuum oven at 100 °C for 12 h before being transferred into the glovebox.

### 2.8 Coin Cells Preparation and Galvanostatic Cycling

Cells were assembled in an argon-filled MBRAUN UNIlab glovebox. The sputtered electrodes were placed in stainless steel 2032 type coin cell (Cambridge Energy Solutions) bases on top of a stainless-steel spacer disc, followed by a glass-fiber separator (Whatman GF/B) which was then soaked with 0.15 mL LP30 electrolyte, a lithium metal chip, another stainless-steel spacer, a spring, and finally the lid. The cells were sealed and rested for at least 10 h. Cells were then cycled on a galvanostatic cycler (LANDT) at approximate rates of C/30 from the open-circuit voltage to 5 mV for the first cycle, followed by approximate rates of C/10 to 100 mV (later 45 mV). The C-rates were calculated using the assumed mass of 28 μg and the theoretical capacity of silicon^1^ of 3579 mAhg^–1^. The applied currents correspond to rates of approximately C/30 in the first cycle and C/10 in the subsequent cycles. There were two cells cycled for each variant.

### 2.9 Data Analysis

Most battery cyclers record the current, time, and voltage of the cell being cycled. The data in this analysis were collected on a Landt potentiostat. By integration of the current with respect to time, the capacity can be obtained. A new data point is normally recorded when a certain time period has passed or a certain change in voltage has occurred, whichever of these conditions is met sooner. When the cell is on a voltage plateau and dV is very small, any uncertainty in the voltage is amplified with respect to the actual change in voltage. To reduce the artifacts that this introduces, a Python script was developed to extract the dQ/dV features. This script was subsequently published on GitHub as part of the Navani Python package.^42^

The script linearly interpolates between data points to normalize the number of data points within a voltage range. A Savitzky–Golay filter smoothing algorithm is applied, and polynomial splines are then fitted to the smoothed data, where capacity is a function of voltage.^43^ These functions are differentiated to give dQ/dV as a function of voltage. An autocorrelation was measured on the resulting smoothed data to assess the noise level. If this was below a threshold value, the initial smoothing was increased, and the differentiation was repeated. There is naturally a trade-off with the level of smoothing and the closeness of fit to the recorded data, so it was attempted to find the least smoothing required to accurately extract the dQ/dV peaks. The resulting dQ/dV is then smoothed again by a Savitzky-Golay filter, and a peak detection algorithm from the analytic_wfm Python package was used to extract the height and location of the dQ/dV peaks within a voltage range.^44^ This enabled the automatic tracking of dQ/dV features over hundreds of cycles for multiple cells.

The positions of the dQ/dV peaks were tracked for each sample over the course of their lifetime with both the potentials and heights of the peaks measured.

### 2.10 Scanning Electron Microscopy (SEM), Energy-dispersive X-ray Spectroscopy (EDS), and Focused Ion Beam (FIB) SEM

Electron micrographs were recorded on a TESCAN MIRA3 with a Schottky field emission (FE) electron gun using a secondary electron detector and an Oxford Instruments X-MaxN with an 80 mm^2^ sensor for EDS analyses. Samples were studied at a working distance of 6 mm at different acceleration voltages, and EDS analysis was carried out at 15 mm WD. FIB SEM was performed on a ZEISS CrossBeam 540 SEM/FIB and an FEI Helios Nanolab SEM/FIB. Samples for FIB-SEM analysis were sputter-coated with gold prior to the transfer into the microscope chamber. In the process, all samples for FIB-SEM analyses were exposed to air. A gallium ion beam was used to deposit a platinum bar from a precursor at the site of interest to limit the damage to the surface during the milling process.

## 3 Results

### 3.1 Polymer Synthesis

The synthetic pathway to the poly­(phosphazenes) used as coatings is laid out in Scheme 1. First, the monomer was synthesized by the optimized synthesis reported by Manners and co-workers from PCl_3_ and freshly prepared LiN­(SiMe_3_)_2_, followed by oxidative chlorination from sulfuryl chloride.^38^ Poly­(dichlorophosphazene) is then formed by adding of a small amount of PCl_5_ to a solution of lithium bis­(trimethylsilyl)­amide, starting the cationic “living” polymerization. This results in short-chain polymers that can later be easily dissolved for both functionalization and the preparation of low viscosity spin-coating solutions, which fill the nanoscopic surface of the Si electrode for the coating process. Functional groups are then added by reacting the P–Cl-bonds with sodium alkoxides. By using an excess amount of sodium methoxy­(ethoxy)­ethoxide solution, a polyether-functionalized polymer **PN­(MEE)** was formed. Adding a less-than-stoichiometric amount of this polyether solution, followed by an excess of a sodium trifluoroethoxide solution, resulted in the formation of a mixed substituted polymer **PN­(CF)**. The polymers were purified by dialysis in water and were estimated to have mass average molecular weights (Mw) of 4.52 × 10^4^ and 1.46 × 10^4^ g mol^–1^ (against a polystyrene standard) and polydispersity indexes of 2.17 and 1.29, respectively, using gel-permeation chromatography (Figure S1). The presence of both functional groups in **PN­(CF)** was confirmed by using MALDI-TOF analysis (Figure S2). The fragmentation pattern suggests that the substitution pattern of **PN­(CF)** is nongeminal where possible, meaning that first a monosubstitution of a chloride phosphorus on each atom of the backbone occurs before the second substitution at the same atom. Therefore, mostly the mixed substitution pattern of both groups at the same atom exists, but both other substitution patterns can be present. The ratio of 1.3 to 0.7 equiv of the different alkoxide side groups was determined by ^1^H NMR spectroscopy (Figure S3).

**Scheme 1:**
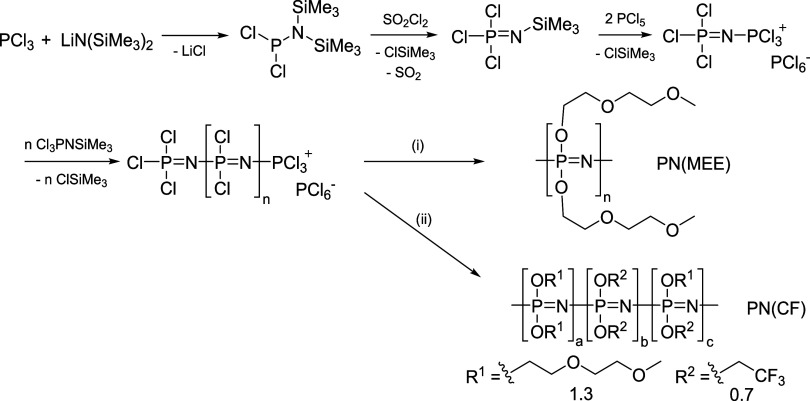
Preparation of the Poly­(phosphazenes) by the “Living” Cationic Polymerization Followed by the Functionalization with Sodium Alkoxides: Either (i) 2.1 equiv NaOEtOEtOMe, THF, 5 d or (ii) 1.5 equiv NaOEtOEtOMe, THF, 2 d + 1.5 equiv NaOCH2CF3, THF, 2 d^a^

a The ratio of the side groups (1.3 equiv *R* ^1^ to 0.7 equiv *R* ^2^, or (2a+c):(2b+c)) in polymer **2** was estimated by ^1^H NMR spectroscopy (Figure S3).

TGA analysis performed in nitrogen gas revealed a high stability of PN­(MEE) to thermal decomposition (TD), with minimal change in sample mass seen below 265 °C (Figure S4), consistent with previous reports.^45,46^ Beyond TD, a sharp decrease in mass is observed (∼40% loss between 265 and 310 °C), likely resulting from initial breakdown of ether linkages in the side chains. Beyond 310 °C, the mass loss rate is reduced, likely resulting from gradual carbonization of the remaining side chains. By the end of the sweep (at 800 °C), the total mass remaining is ∼18.9% of the starting mass. If the entire etheric side chains were lost, leaving only PN species behind, a residual mass of 16% is predicated. This suggests that the observed mass changes are likely a result of side-chain thermal decomposition and partial skeletal oxidation (from either dissolved oxygen in the sample, or etheric side-chain oxygens), potentially forming some phosphate or polyphosphate ceramic-type materials. TGA crucibles showed a black solid at the end of the experiment, which was insoluble in concentrated HCl, suggesting that some P and N-doped carbons may be present. The high thermal stability of poly­(phosphazenes) has led to prior work investigating phosphazene-based materials as flame retardant/extinguishing components inside batteries, potentially alleviating the safety concerns of the use of flammable solvents and plastic separators.^47,48^ Tests using a lighter did not lead to ignition of the bulk polymers, demonstrating their flame retarding qualities (Figure S5).

### 3.2 Electrode Preparation

The different stages of electrode preparation by sputtering are shown in Figure 2a–c. The cold-rolled copper current collector has a microscopically rough surface that is evenly coated with the nickel and silicon metal (Figure 2d). The color of the electrodes varies due to slight variations in thickness. Next, the sputtered anodes were spin-coated with solutions of either **PN­(MEE)** or **PN­(CF)** (10 wt % in toluene) and benzophenone (2 wt %) as a cross-linking agent. After drying, the coated samples were exposed to UV light for cross-linking; this had a noticeably better effect for **PN­(MEE)** compared to the **PN­(CF)** films since the mechanism is a radical-activated reaction that should benefit from more available hydrogen–carbon bonds on the polyether-side chains. When applying poly­(phosphazenes) bearing only −OCH_2_CF_3_ in a separate trial, no film was formed under the same curing conditions. The coatings formed from the partially fluorinated **PN­(CF)** were sticky to the touch even after UV-treatment and showed a significantly lower water contact angle of 12.0 vs 68.3° for the uncoated and 85.2° for the **PN­(MEE)**-coated electrodes (see Figure S6), despite the high degree of fluorination. Focused ion beam-milled cross sections show that the polymers form a coating of ≳100 nm in thickness, filling the grooves in the surface morphology (Figure 2f), with occasional minor defects (Figure 2g, red circle). **PN­(MEE)** coats the electrode evenly (Figure 2g), while **PN­(CF)** is more inhomogeneous, likely due to the different substitution patterns and hence physical properties of the polymer fractions (Figure 2h, yellow circle).

**Figure 2:**
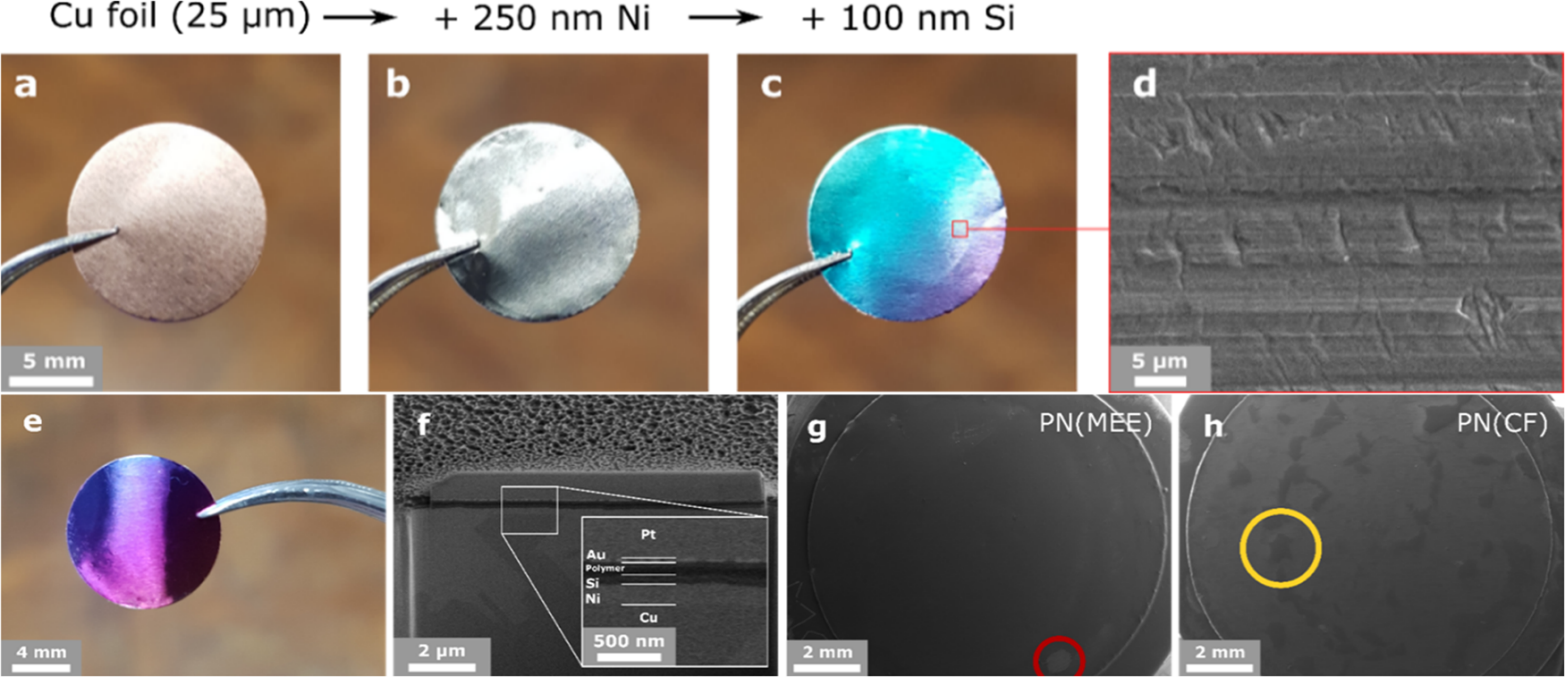
Photographs of the different stages during the preparation of the silicon thin-film anodes: (a) current collector, (b) coated with 250 nm nickel, (c) coated with 250 nm nickel and 100 nm silicon. (d) SEM micrograph of the silicon and nickel-coated electrode. (e) Poly­(phosphazene)-coated Si thin-film electrode. (f) FIB SEM micrograph of the coated electrode. Layers from bottom to top: current collector, 250 nm Ni adhesive layer, 100 nm Si active material, poly­(phosphazene)-coating, thin sputtered gold layer and 1 μm thick deposited platinum bar for FIB purposes. SEM micrographs of a coated electrode with (g) **PN­(MEE)**, (h) **PN­(CF)**. Red and yellow circles indicate regions with irregularities. Adapted with permission from ref 49. Copyright 2020 N.-J. H. Kneusels.

### 3.3 Electrochemical Cycling

The poly­(phosphazene)-coated silicon thin-film electrodes were cycled against lithium metal in coin cells using a standard carbonate-based electrolyte (LP30) using galvanostatic methods (Figure 3). Duplicates can be found in Figure S7 in the SI. A cutoff voltage of 100 mV was used, which does not allow the fully lithiated state to be reached, reducing the compressive and tensile stress on the material due to a smaller volume expansion during lithiation and delithiation. To allow comparison between films and typical Si electrodes the measured capacity was converted to mAhg^–1^, using an estimated mass of 28 μg and a specific capacity of 3579 mAhg^–1^.

**Figure 3:**
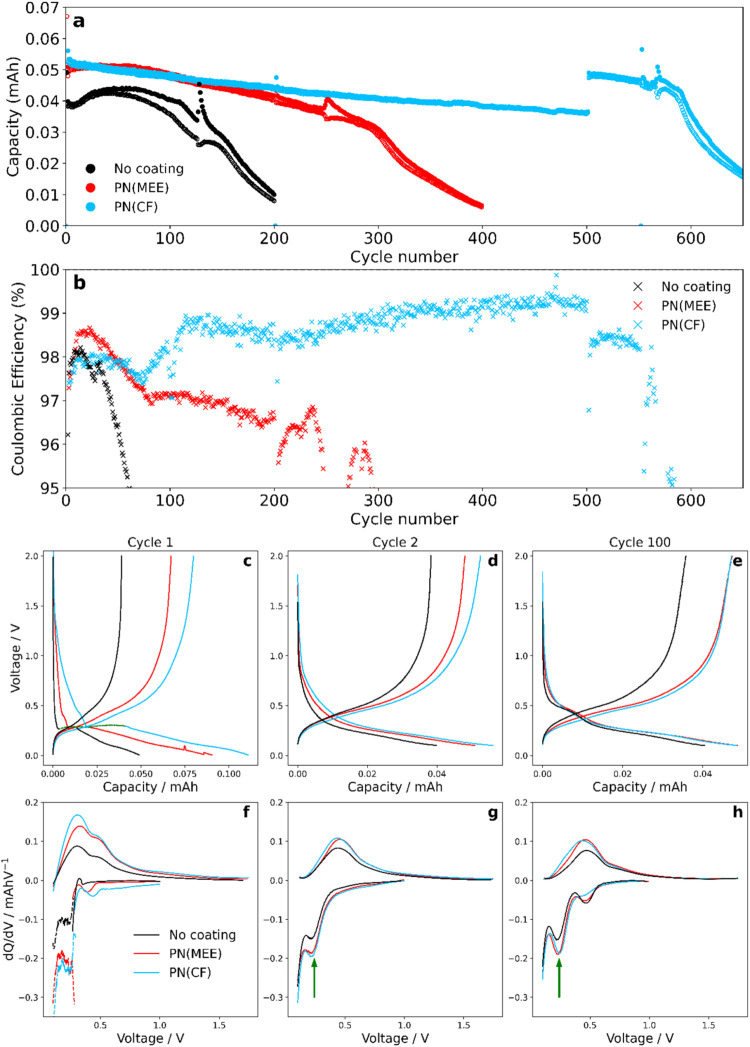
Electrochemical performance of 100 nm silicon thin-film electrodes without, and with **PN­(MEE)** and **PN­(CF)**-coatings; the silicon “anodes” were cycled vs Li metal and thus discharging corresponds to lithiation of the silicon film. A cutoff voltage of 5 mV and a slow rate (C/30) was used for the first formation cycle; cycling was performed between 2.0 and 0.1 V at rates of C/10 thereafter. (a) Lithiation (upper trace) and delithiation capacities (lower trace) and (b) Coulombic efficiencies for the first 500 cycles. All cells were set to 200 cycles; stable cells were then restarted and cycled for a further 450 cycles. (c–e) Lithiation and delithiation profiles of the 1st, 2nd and 100th cycle of the three anodes. (f–h) Differential capacity plots of the respective cycles showing the electrochemical processes in more detail. The ∼0.23 V process assigned to the formation of a-Li_2_ Si is marked with a green arrow. In panel (c), the voltage increases during the plateau at ∼300 mV, this region has been plotted in green and was removed from the dQ/dV analysis.

The uncoated electrode has about 80% of the capacity of the coated electrodes, which we ascribed to variations in the amount of Si deposited in the sputtering process and thus errors in the calculation of the gravimetric capacity. All three half cells show a low first-cycle CE: 79.4% for the uncoated electrode, 74.3% for the PN­(MEE)-coated electrode, and 72.0% for the PN­(CF)-coated electrode. This is in line with other studies on silicon thin-film electrodes; for example, work by Swallow et al. reported CEs of around 70%.^50^ This large first-cycle irreversible capacity is attributed to irreversible restructuring of *a*-Si and the large surface area of the thin film, which promotes electrolyte reduction, SEI formation and a greater contribution from the reduction of the surface passivation layer, SiO_2_. In this study, there may also be trace amounts of water in the polymer layer, which could further contribute to the low initial CE. These effects become clearer in the high voltage region of the cycle (Figure S8). To put the surface effects into perspective, we found that thicker films show better Coulombic efficiency in the first cycle but are more prone to early delamination and fade more quickly (Figure S9). The CE then increases over the first few cycles (Figure 3b), as a more passivating SEI is formed. The uncoated electrode maintains its maximum capacity for about 100 cycles before a steep reduction in specific capacity and CE is observed. Over the course of cycling, the cell only reaches 98.2% in CE, which is then followed by a gradual decrease. However, at around cycle 127, the capacity shoots up and the CE decreases; after this spike, the cell capacity rapidly fades. The spike is likely caused by delamination of the Si film from the substate. The **PN­(MEE)** coated electrode shows a similar trend, but the spike and then steep drop in capacity and CE occurs only after reaching more than 200 completed cycles, and the efficiency is ultimately higher and fades at a notably slower rate compared to the reference. The **PN­(CF)** coated electrode shows similar CEs over the first 80 cycles to the other electrodes after which it increases, surpassing CEs of 99.4% and no spike or sharp drop-off in capacity is seen even after 500 cycles. To probe the degradation of the **PN­(CF)** coated electrode, additional stress was simulated on the electrode by lowering the cutoff voltage to 45 mV after 500 cycles, allowing a greater extent of lithiation of the silicon anode (Figure 4). This yields an increase in capacity by 500 mAhg^–1^ and no additional capacity fade is observed during the following 50 cycles; however, the CE decreases, suggesting that more irreversible side reactions take place in the larger voltage window. After resting for a short while and further cycling, a degradation process like that observed for the uncoated and **PN­(MEE)**-coated anodes as shown in Figure 3a,b eventually appears to take place.

**Figure 4:**
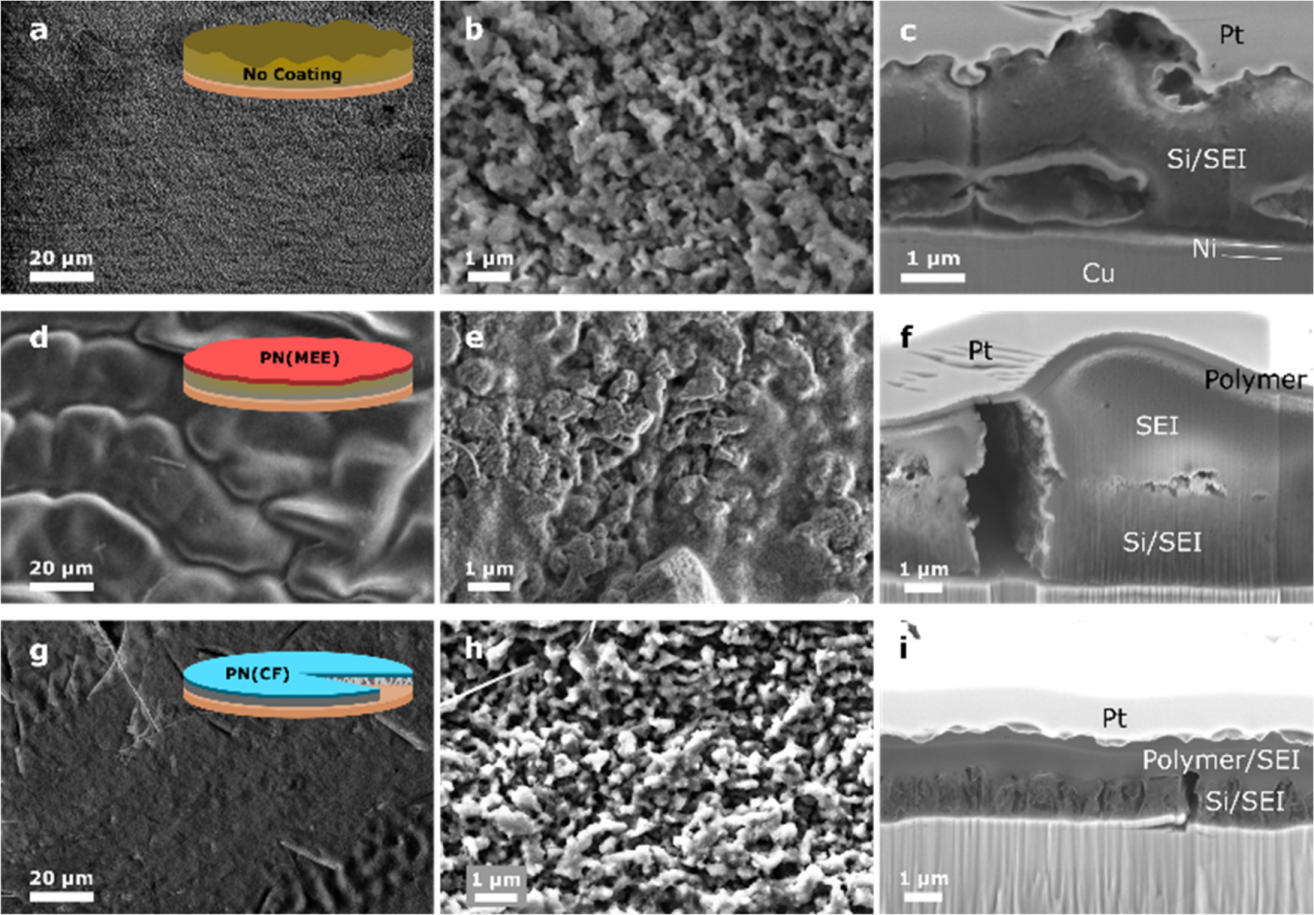
SEM micrographs of the cycled silicon thin-film electrodes. (a–c) Without a coating after 200 cycles; (d–f) a **PN­(MEE)** coating after 500 cycles; (g–i) a **PN­(CF)** coating after 525 cycles; panels (d) and (g) show the films with the polymer coatings intact; panels (e) and (h) show the surface underneath the pealed-off polymer layer to see the Si microstructure. Panels (c), (f), and (i) are FIB cross sections prepared following the deposition of a platinum bar. The individual phases in each electrode are labeled based on the EDS data shown in Figure 5. Adapted with permission from ref 49. Copyright 2020 N.-J. H. Kneusels.

The lithiation-delithiation profiles and differential (dQ/dV) plots are shown in Figure 3 for cycles 1, 2, and 100. The pristine material (cycle 1) shows a sharp delithiation plateau at ∼300 mV that is not present in subsequent cycles. This peak is common in silicon thin-film electrodes and has been ascribed to an irreversible rearrangement of the Si structure to form a lithiated amorphous silicide.^50^ In subsequent cycles this feature is no longer present, likely because a more open *a*-Si phase has formed, with more lithiation pathways, on delithiation. All subsequent cycles show a distinct dQ/dV lithiation peak at 250 mV corresponding to the formation of the first amorphous “phase” Li_2_Si. The capacity below 175 mV corresponds to the onset of the second lithiation step toward a composition of Li_3.5_Si.^16,51^ A dQ/dV peak at 450 mV seen clearly in cycle 100 is tentatively attributed to the degradation of the electrolyte, as it appears in the formation cycle and then reappears in later cycles during degradation of the electrode and capacity fading. By the 100th cycle, the uncoated and **PN­(MEE)**-coated cells show a significant increase in the 450 mV peak height compared to the **PN­(CF)**-coated cell. The increase in peak intensity also corresponds to decreased Coulombic efficiency, further hinting toward a mostly irreversible degradation mechanism.

### 3.4 Electron Microscopy and Elemental Analysis

Electron microscopy of the uncoated electrode and the **PN­(MEE)** coated electrode was carried out after 200 and 500 cycles, respectively. Upon disassembly of the cells, the electrodes appeared pale brown having lost the metallic color due to the increase in thickness. It should be noted that these cells were electrochemically dead, and for the **PN­(MEE)** coated electrode, the cell was functionally dead after 400 cycles, i.e., 100 cycles before the analysis were done. In contrast, the **PN­(CF)** coated electrode was analyzed after 525 cycles and taken from a functional cell. Micrographs of the electrode surfaces and the respective cross sections in Figure 4 show significant structural changes of the electrodes from cycling. The uncoated Si electrode has expanded considerably, as the focused ion beam (FIB) cross-section reveals a massive expansion of the electrode to thicknesses of 2 to 4 μm from the original 100 nm thick film. Furthermore, the film has developed a rough nanostructure, as seen from the top (Figure 4b), with large cavities forming a sponge-like material, which still contains electrolyte after drying. The **PN­(MEE)** coating has swollen noticeably into a wrinkled surface layer (Figure 4d). It has two to three times its original thickness (Figure 4f) and covers the electrode without any visible holes. Underneath it, in Figure 4e, electrolyte and material formed from electrolyte decomposition has accumulated. The FIB cross-section in Figure 4f shows the formation of a thicker composite, compared to the bare Si reference electrode, that appears more porous in regions close to the current collector and shows a smoother texture in areas directly underneath the polymer coating. The bottom layer is as thick as the uncoated electrode’s Si-SEI composite, suggesting a similar structural change of the Si material. The wavy top layer is between 1 and 5 μm thick. Electrochemically inactive electrode material has partially delaminated near vertical cracks. These are likely caused by the mechanical stresses from shrinking during drying. In contrast, the **PN­(CF)**-coated electrode is mostly flat (Figure 4g) and shows a different structure in its cross-section. Strands of the Si-SEI composite have formed and are attached to the current collector. This layer of strands is only half as thick as the other electrodes’ structures. Interestingly, the Ni interlayer is seen to delaminate from the Cu, rather than the composite from the Ni (additional micrographs of the phenomenon are presented in Figure S10), suggesting that the Si is still firmly attached to the nickel.

EDS analysis (Figure 5) was used to investigate the chemical composition of the electrodes with specific interest in Si-rich and P-rich areas as well as the distribution of the typical elements commonly found in the SEI (C, O, F). The signal from the Ni adhesion layer serves as an indicator for the electrode material distribution and density as nickel is more visible where there are voids and cracks, the EDS Ni signal coming from the substrate below rather than the film itself. The analysis was carried out 1 day after the FIB SEM cross-sectioning, leading to some electrolyte leaching from the electrode and drying on the surface (uncoated electrode, Figure S11). Residual electrolyte was identified by an increase in signals from oxygen and carbon (i.e., carbonates) and fluoride (degradation products from LiPF_6_). The polymer **PN­(MEE)**-coated electrode elemental maps show a separation between the two layers of Si-rich SEI composite and additional SEI below a smooth top layer (Figure 5b). Si is detected only in the bottom layer, and an increased amount of O, C, and F is seen in the top layer. It is difficult to distinguish the polymer from the SEI, as P from both LiPF_6_ and its degradation products is present in all layers. In Figure 5c, the layer formed from **PN­(CF)** can be distinguished from the Si layer with significant amounts of F and P detected. This may be in part attributed to the polymer containing −CF_3_ groups but again mostly to LiPF_6_. Large amounts of both O and C are in the layer formed from **PN­(CF)** and a small amount in the Si-SEI strands underneath. Si is found at high concentrations in the vertical strands. Additionally, the strong Ni signal suggests that there are void spaces between the strands, with one such large void or crack being clearly visible in the SEM image, the crack penetrating throughout the film thickness. These results should be contrasted with our recent study of uncoated and thinner silicon film electrodes, where operando X-ray absorption spectroscopy was used to show that the inner layer of the SEI contains larger amounts of LiF, with the outer layer containing more organics.^50^ However, in our ex situ studies of cycled electrodes, the SEI components become more difficult to distinguish from the electrolyte that has dried and decomposed underneath the polymer layer.

**Figure 5:**
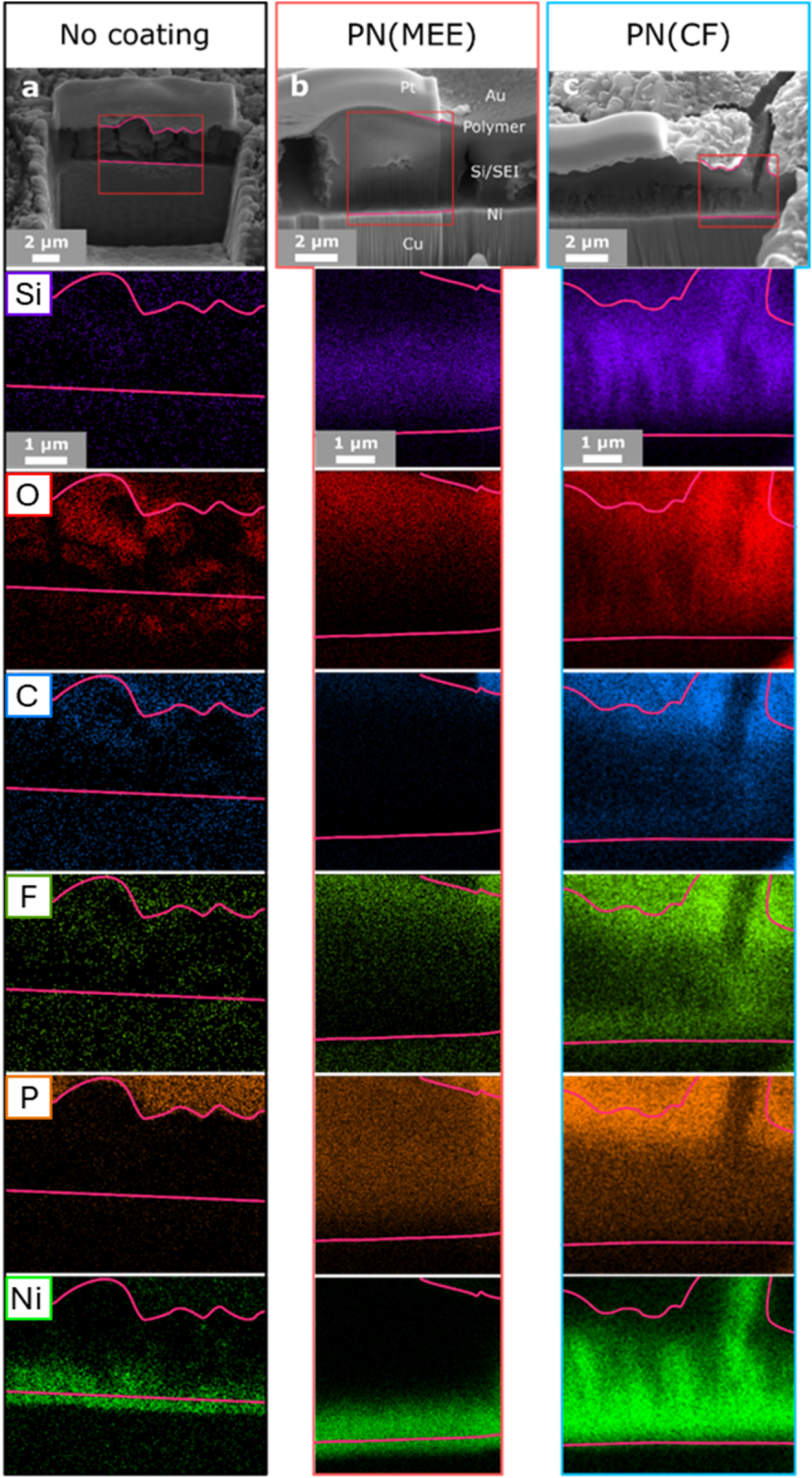
SEM micrographs and EDS element maps (15 kV) of the FIB cross-section of the 100 nm *a*-Si thin-film anodes on Ni-coated copper with and without poly­(phosphazene) coatings. (a) Electrode without a coating after 200 cycles. (b) **PN­(MEE)** coating after cell failure (500 cycles). (c) **PN­(CF)** coating after 525 cycles. The surface was coated with Au and a Pt bar was deposited. The highlighted areas in the micrographs correspond to the EDS elemental maps with marked borders enclosing the area between the platinum bar and the nickel interlayer. Adapted with permission from ref 49. Copyright 2020 N.-J. H. Kneusels.

## 4 Discussion

Three Si thin-film electrodes were cycled, one reference without a coating, and two with different poly­(phosphazene) coatings. The coatings have altered the electrodes’ electrochemistry differently, with positive results in both cases. **PN­(MEE)** extends the lifetime significantly but shows similar degradation of the silicon material to the uncoated electrode; **PN­(CF)** extends cycle life even further and provides superior cycling properties. In all three cases, the extent of SEI formation is still significant, with the films growing to around 1–5 μm from their original thickness of 100 nm. The uncoated electrode has grown to approximately 2–3 μm after 200 cycles. The **PN­(MEE)** coated sample has grown to approximately 3–5 μm after 500 cycles, although, as noted earlier, the last 100 “cycles” are of very low capacity, and the electrode can be considered dead by 400 cycles. The **PN­(CF)** coated sample shows the slowest growth, reaching a thickness of 2–3 μm after 525 cycles.

The total capacity of the three samples varies, not only due to the stabilization by the coatings but also due to the variations of the total silicon deposited during the sputtering process. Nevertheless, in the early cycles, a similar CE is observed. All cells have been cycled to the same cutoff voltage of 100 mV at first, limiting the available capacity, but allowing longer cycling due to less stress from volume expansion during lithiation, and potentially less reduction of the electrolyte to form SEI.^11,12^ The differential plots in Figure 3c–g show that only the first lithiation stage is fully achieved at approximately 250 mV, which corresponds to the formation of *a*-Li_2_Si from Si, and that the second “phase” transition from *a*-Li_2_Si to *a*-Li_3.5_Si that occurs at approximately 100 mV is only just beginning to start but is not finished. Going to a lower cutoff voltage such as 75 mV will form more of the *a*-Li_3.5_Si phase, leading to increased capacity but also increased volume expansion and degradation. Lowering the cutoff voltage even further will allow the formation of the crystalline Li_3.75_Si-phase, vastly accelerating degradation.^12^ However, calculations have shown that in a full-cell setup using state-of-the-art cathode materials, anode capacities beyond ∼2000 mAhg^–1^ may not significantly increase the overall gravimetric capacity of the full cell due to the low intrinsic gravimetric capacity of the cathode material.^52^ Therefore for silicon it may not be required to go to such low cutoff voltages. Voltage limitation therefore should in general allow for good long-term cycling of the material, avoiding excessive degradation due to volume expansion, while still being viable for prospective applications.

Compressive and tensile stresses play a significant role in the silicon thin-film electrode thermodynamics during lithiation and delithiation, respectively. There is a stress-potential coupling when forming lithium silicide on a thin-film substrate, as demonstrated by Sethuraman and co-workers,^14,15^ with higher stress lowering the potentials of lithiation (vs Li). The major peak (formation of *a*-Li_2_Si) seen in the dQ/dV plots is further analyzed in Figure 6 to explore this. The height is a measure of the flatness of the voltage “plateau” or lithiation process. The peaks corresponding to the formation of the *a*-Li_2_Si “phase” (green arrows in Figure 3g,h) in both potential and height are plotted in Figure 6a,b. The overpotential of the cell was also measured by taking the difference in the potentials of the formation and decomposition of *a*-Li_2_Si in lithiation and delithiation. This is plotted in Figure 6c. Purely thermodynamic effects such as the stress-potential coupling should not change this overpotential, whereas kinetic effects such as SEI formation will.

**Figure 6:**
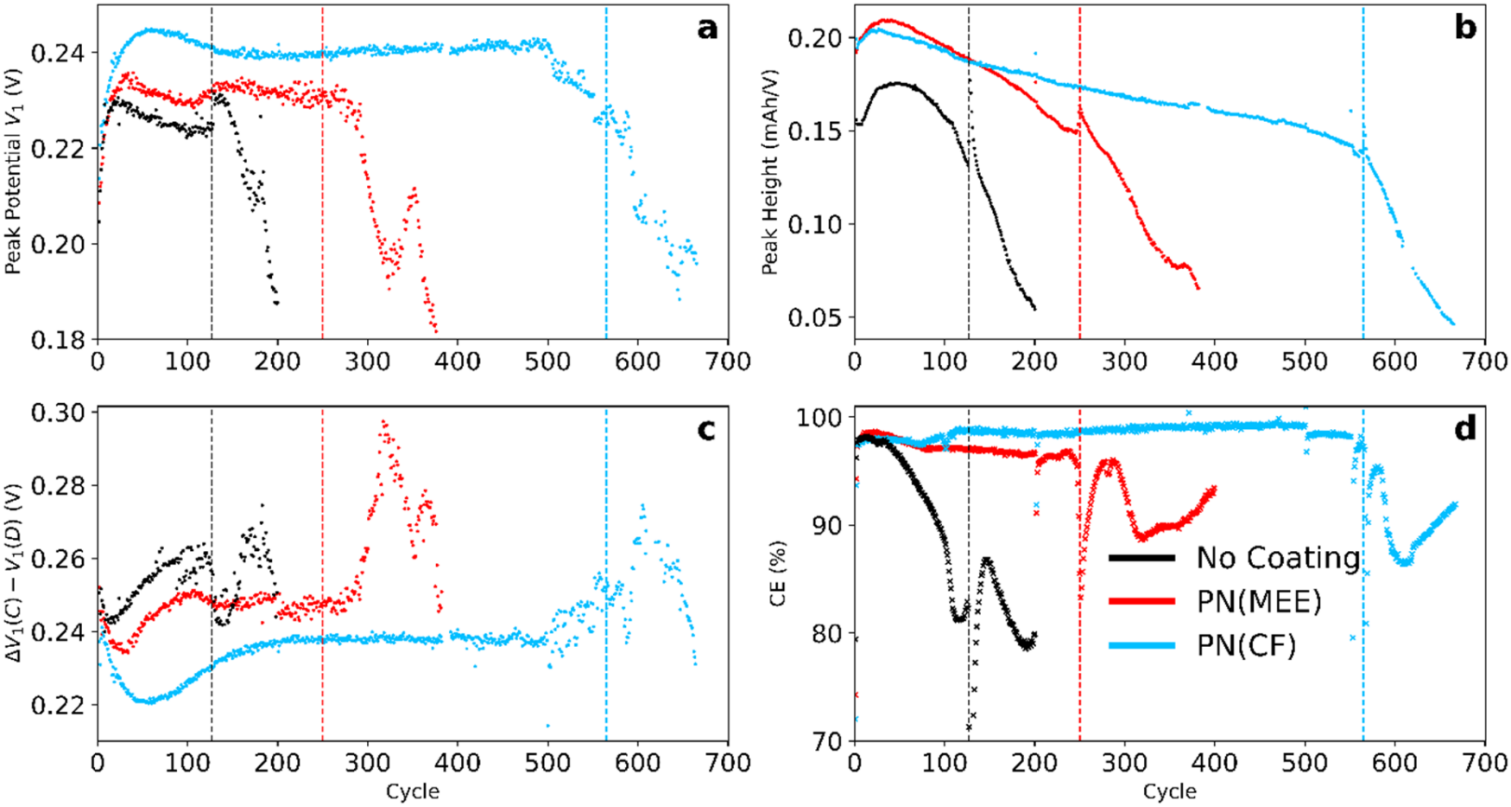
Electrochemical signatures of half cells with the three types of silicon anodes: the uncoated anode shown in black, the **PN­(MEE)**-coated cell in blue, and the **PN­(CF)**-coated cell in red. (a) Voltage and (b) height associated with the *a*-Li_2_Si formation process during lithiation (∼0.23 V); (c) the overpotential, defined as the difference in potential between Li_2_Si formation and decomposition between lithiation and delithiation, and (d) the Coulombic efficiency vertical lines indicate the characteristic “dips” in efficiency, attributed to delamination of the Si layer from the copper substrate.

A possible stress-potential coupling becomes evident in the first few cycles by the slow increase in potential of the lithiation processes. All three films start in the first cycle by forming *a*-Li_2_Si at a similar voltage of 0.22 V and show an increase over the next few cycles to 0.23 V, as shown in Figure 6a. This increase in lithiation potential corresponds to a decrease in the overpotential (Figure 6c), measured by comparing the potential of the lithiation and delithiation process in charge and discharge. The cause is likely to be the cracking of the material and opening lithiation pathways in the amorphous film reducing stress across the electrode and reducing the activation barrier for lithiation. As the material separates further into small islands, the potential rises. The formation of such island-like structures is well-known for Si thin films. By 0.23 V, the uncoated electrodes and those coated with **PN­(MEE)** have reached a maximum for the stable cycling period. After the Si electrode is sufficiently cracked a decrease in the lithiation potential and an increase in the overpotential are seen, these are likely to arise from the continuous formation of SEI, increasing the impedance and negatively affecting the electrodes kinetics. However, the **PN­(CF)** coated electrode reaches a higher lithiation potential of 0.25 V, and a lower overpotential of ∼0.22 V. The increase in the electrode potential could be explained by lower stress across the electrode, leading to lithiation being thermodynamically favorable at higher potentials, or the **PN­(CF)** coating affecting the nature of the SEI, supporting easier lithium-ion transport and better electrode kinetics. The lower overpotential points to there being at least some contribution from improved kinetics.

Tracking the trends observed in the lithiation potential, there are strong similarities between the uncoated and **PN­(MEE)** coated electrodes. All of the cells exhibit an abrupt change that follows a “dip” in Coulombic efficiency as seen labeled in Figure 6 by a dashed line. A rapid drop in capacity then occurs shortly after, which is accompanied by a gradual drop in potential of the “0.22 V” lithiation process. As previously reported, typical degradation of a Si thin-film anodes proceeds through stages of cracking, pulverization, and delamination.^1,12^ The increase in overpotential (Figure 6c) indicates that the drop in capacity is kinetic in origin, and likely relates to poorer electrical contact to the Si associated with the onset of the delamination process.

The height of the lithiation feature associated with the formation of *a-*Li_2_Si (Figure 6b) shows a slow decrease as the capacity of the cells fade for all samples, with jumps in height at the delamination events marked by the vertical dashed lines. Following the potential at which *a*-Li_2_Si formation occurs, offers a useful tool to trace the electrode’s degradation. In this case it has even allowed us to make suggestions on the possible degradation mechanism of the poly­(phosphazene) coated silicon thin-film electrodes. Taking the electrochemical and FIB-SEM results into account, we can describe two different mechanisms of stabilization of a Si thin-film anode involving poly­(phosphazene) coatings, depicted in Figure 7. The **PN­(MEE)-**coating is more efficiently cross-linked and provides mechanical stability. As a result, the coating does not show heterogeneous swelling and structural changes during cycling, judging by the relatively uniform thickness observed in the FIB SEM image in Figure 4f. In contrast, **PN­(CF)** had less rigid mechanical properties. When handling the electrode, the surface was still sticky, suggesting a lower degree of cross-linking.

**Figure 7:**
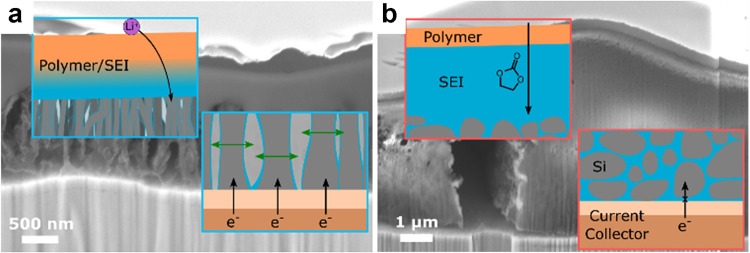
Proposed stabilization and degradation mechanisms of the poly­(phosphazene)-coated silicon thin films used in this work. (a) Mechanical stabilization of the surface and formation of a more stable SEI providing a partial barrier to the electrolyte and supporting a freely expanding Si-structure with effective electronic contact to the current collector. (b) Ultimately delamination and cell failure occur, through excessive volume expansion and contraction and SEI formation after prolonged cycling. Adapted with permission from ref 49. Copyright 2020 N.-J. H. Kneusels.

In the case of a working electrode with a **PN­(CF)-**coating, as portrayed in Figure 7a, Li-ions may still pass through the polymer/SEI layer and form lithium silicides, with the lower Si layer forming strands that are still connected to the current collector. As seen in Figure 4i, these strands appear to still be attached to the polymer/SEI layer, forming a connected structure with the coating and current collector. On cycling the coated electrode, the initial **PN­(CF)**-coated silicon assembly evolves into a porous structure that has subcutaneous voids that can accommodate the large volume expansion caused by lithiation over many cycles. This hybrid SEI/polymer arrangement is fragile, as deeper lithiation (by lowering the cutoff voltage) leads to more volume expansion that eventually disturbs the balance and ultimately leads to delamination and degradation.

Upon degradation and cell failure, Figure 7b shows the inactive Si electrode (in this case coated with **PN­(MEE)**) postcycling and the implications of the structural changes that have occurred on its electrochemistry. The nonfluorinated polymer coating has allowed large amounts of electrolyte to pass and form a decomposed or dried out layer of electrolyte on top of the Si-SEI composite.This is seen in the EDS analysis (Figure 5b), where a high O and F signal layer with little Si signal is present closer to the surface of the sample. The thickness of the Si-SEI composite in the layer closer to the current collector and the similarities in lithiation potential suggest similar degradation mechanisms to those for the uncoated anode. Upon further fracturing and pulverization of the electrode, more SEI will form underneath the Si and lead to delamination, cutting off conductive pathways for electrons from the current collector. Overall, the lifetime of the cell using the **PN­(MEE)** polymer coating is still improved compared to the uncoated sample, probably due to mechanical stabilization that may distribute the forces from volume expansion and stabilize the electrodes’ electrochemistry by keeping the active material in place. Electrolyte permeation is also likely slowed to some extent.

## 5 Conclusions

Poly­(phosphazene) coatings were successfully applied on Si thin-film anodes adding cycling stability and increasing their performance. The thin-film Si anodes were mechanically stabilized by the flexible poly­(phosphazene) coatings increasing their lifetimes. Additionally, by tailoring the side groups of the poly­(phosphazene) coating, in the case of **PN­(CF)**, an SEI is formed that maintains a better integrity during cycling, as shown by the higher Coulombic efficiencies throughout the cell’s lifespan. The Si nanostructure formed during cycling in the coated structures was porous and provided enough space to accommodate the volume expansion during lithiation, enabling stable cycling for over 500 cycles: under similar cycling conditions, stable cycling was extended more than 3-fold when applying the **PN­(CF)**-coating compared to the uncoated electrode, while the fully polyether-substituted **PN­(MEE)**-coating granted a doubling of the expected lifetime. From the two different poly­(phosphazene)-coatings, the differences in the electrode structures observed postcycling and the electrochemical behavior showcase their potential uses when being applied in electrochemical cells. The results also highlight remaining challenges in silicon systems: if coatings are permeable to the electrolyte, SEI formation continues resulting in large swelling of the silicon-SEI composite. Even in thin films, the swelling and mechanical stresses are so large that delamination occurs so that electrical contact is lost.

A careful analysis of the electrochemical signatures of the (de)­lithiation processes was performed, and while illustrating the wealth of information present in a charge/discharge curve, this also highlights the challenges of processing these data effectively, especially when there are large amounts of cycles and the data are noisy. A code was developed by the authors to extract this information, which has been released in the Navani Python package to aid others performing similar analyses.^42^

Given the possibility to introduce an almost unlimited number of side groups into the polymer from different alcohols or amines, this solution-based approach to poly­(phosphazene) coatings provides a versatile tool for designing improved SEIs on silicon thin-film electrodes. Furthermore, this method can potentially be expanded to other anode or cathode thin-film electrodes in batteries, with future work exploring the role that different additives have in improving the Si nanostructures formed in these thin-film systems.

## Supplementary Material



## Notes

Detailed analytics of the polymer synthesis; additional electrochemical cycling data; additional scanning electron micrographs (PDF)
