# Supplementary material for: Poly(phosphazene)-Coatings for Stabilizing Silicon Thin-Film Anodes in Lithium-Ion-Batteries
Source: ACS Appl Mater Interfaces. 2026 Jun 1;18(22):31301–14. doi: 10.1021/acsami.6c04115 (PMC13266691; doi:10.1021/acsami.6c04115)
Supplement: Supplementary file 1 [file am6c04115_si_001.pdf]

Supporting Information

Poly(phosphazene)-coatings for Stabilising Silicon Thin-film Anodes in Lithium-Ion-Batteries

*Nis-Julian H. Kneusels, Ben E. Smith, Kieran Mylrea, Yanting Jin, Zachary Ruff, Robert S. Weatherup, Dominic S. Wright, Clare P. Grey**

The Yusuf Hamied Department of Chemistry, University of Cambridge, Lensfield Road, Cambridge CB2 1EW, U.K.

Email: cpg27@cam.ac.uk

Supporting Figures

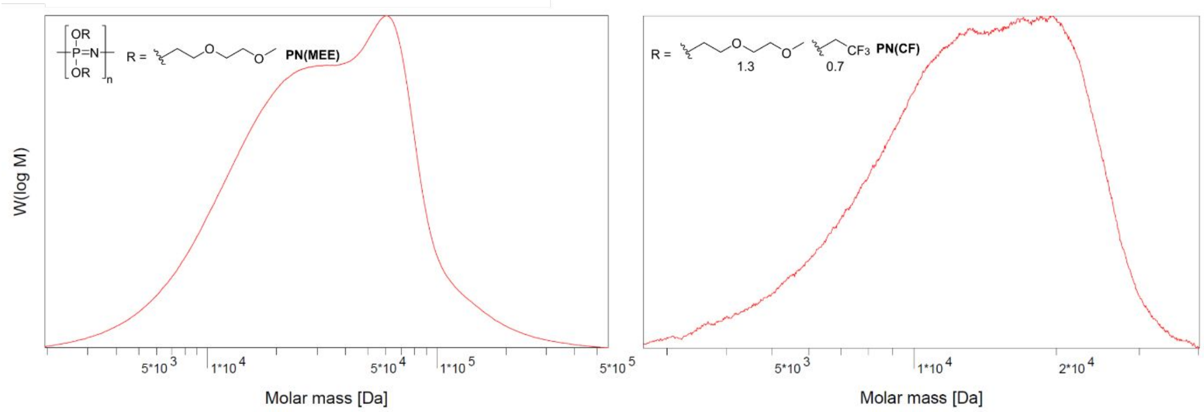

Figure S1: GPC results of PN(MEE) and PN(CF) in DMF. Reproduced with permission.[1] Copyright 2020 N.-J. H. Kneusels.

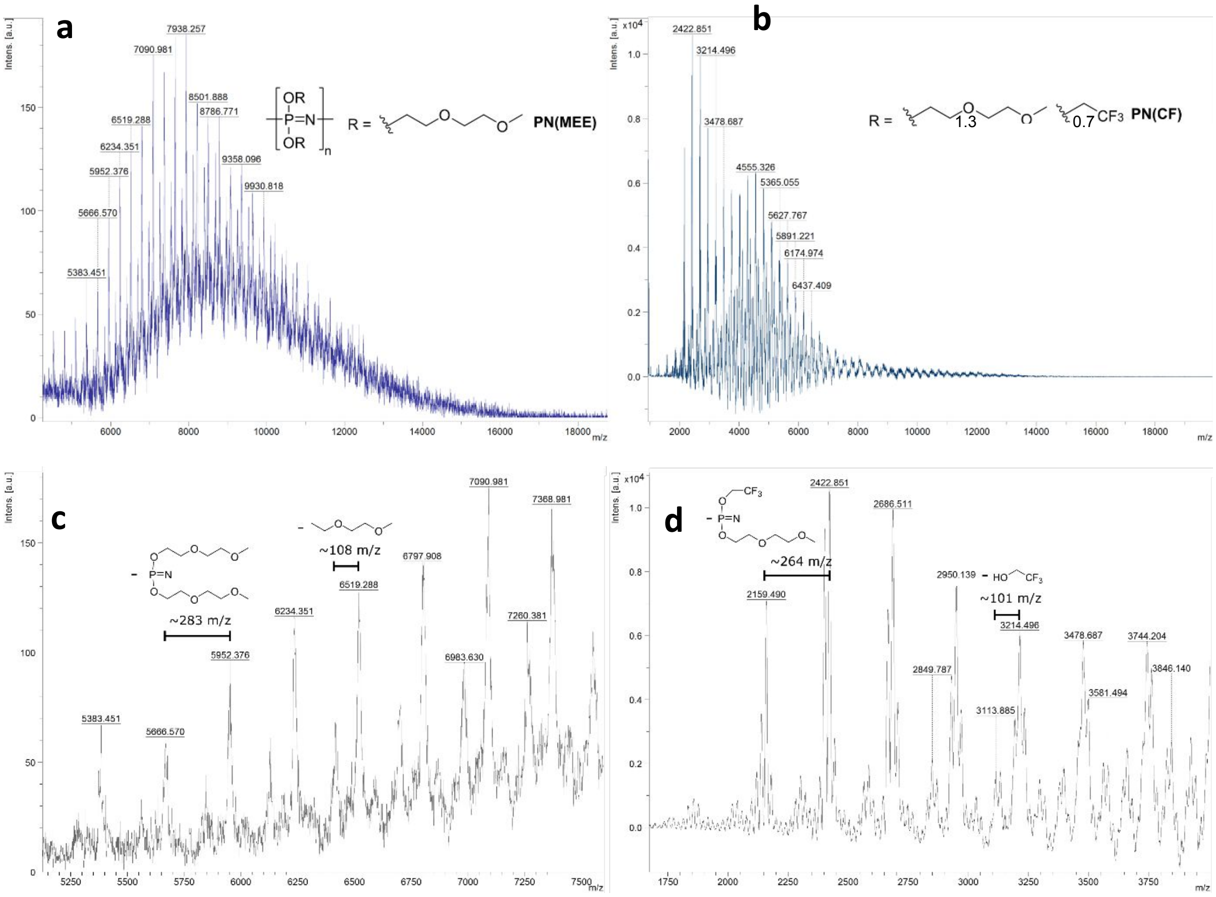

Figure S2: MALDI TOF spectra of a) **PN(MEE)** and b) **PN(CF)** with zoomed in excerpts of the respective spectra in c) and d) showing the respective fragmentation patterns and corresponding phosphazene fragments. Reproduced with permission.[1] Copyright 2020 N.-J. H. Kneusels.

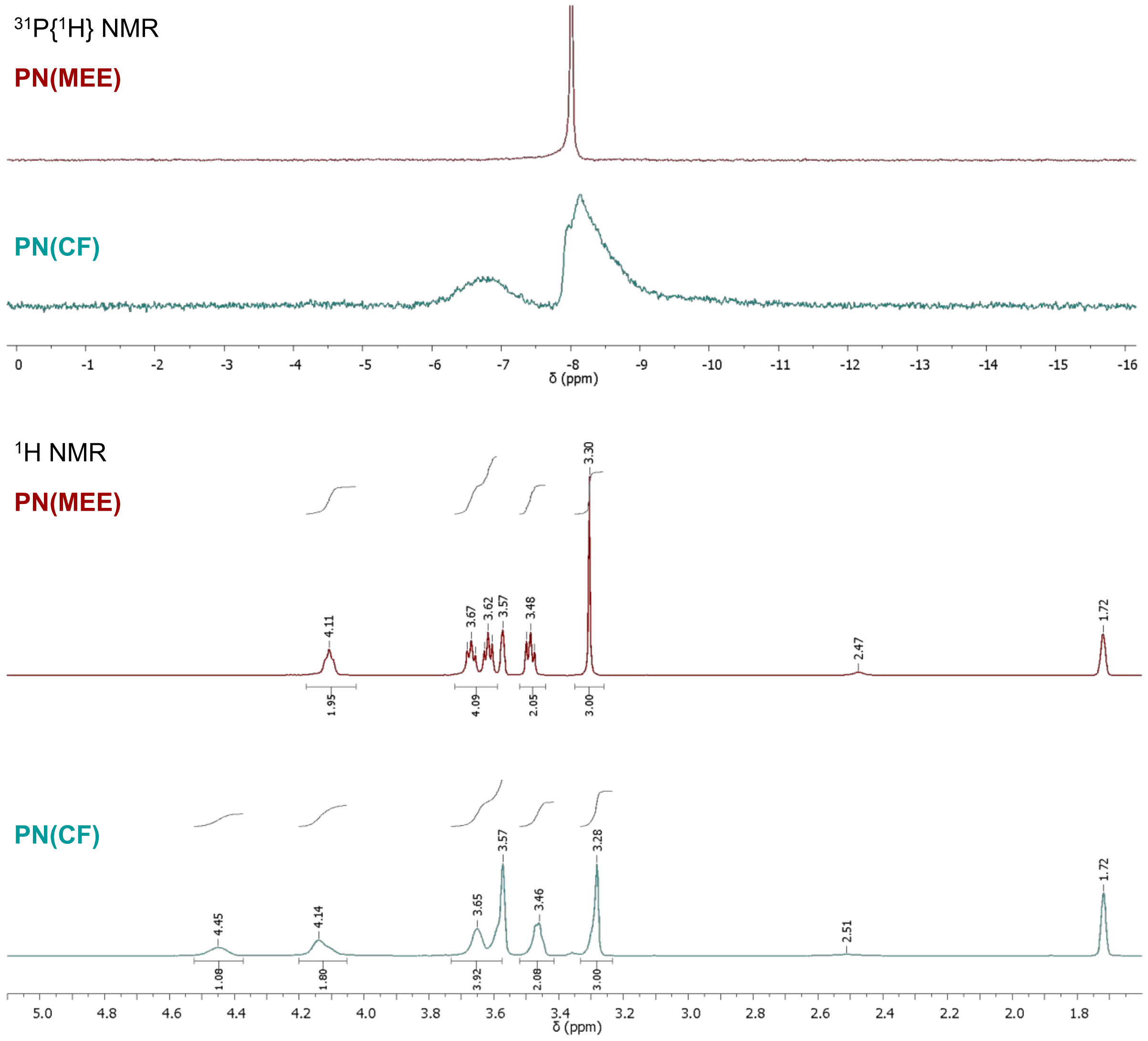

Figure S3: ^{31}P and ^{1}H NMR spectra (THF-d_8, 400 MHz) of **PN(MEE)** (red) and **PN(CF)** (turquoise). Residual proton signals from the THF solvent are detected at 1.72 ppm and 3.57 ppm and traces of water are present at ~2.5 ppm. Reproduced with permission.[1] Copyright 2020 N.-J. H. Kneusels.

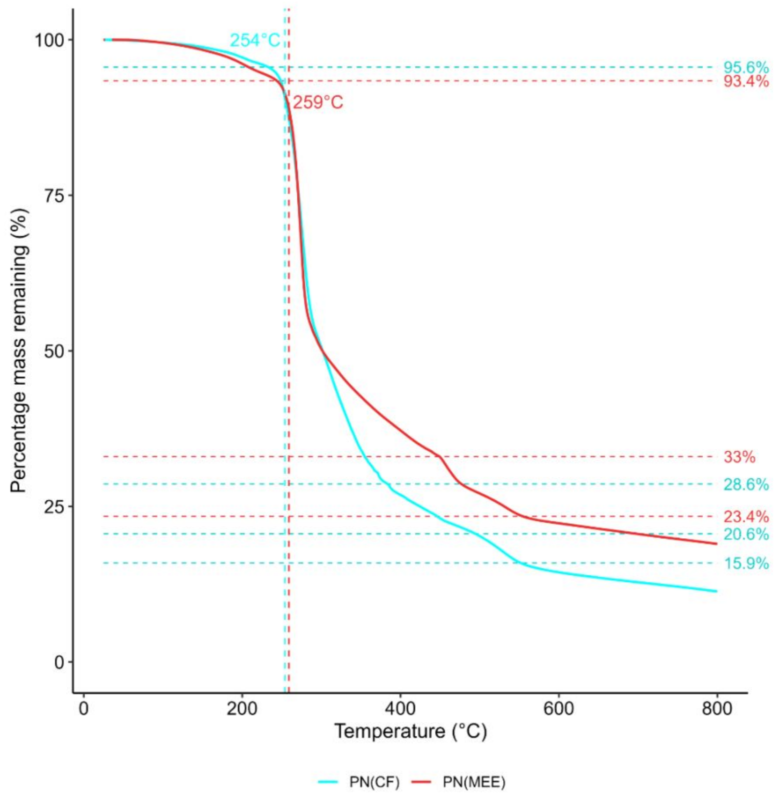

Figure S4: TGA analysis of PN(MEE) (blue) and PN(CF) (red).

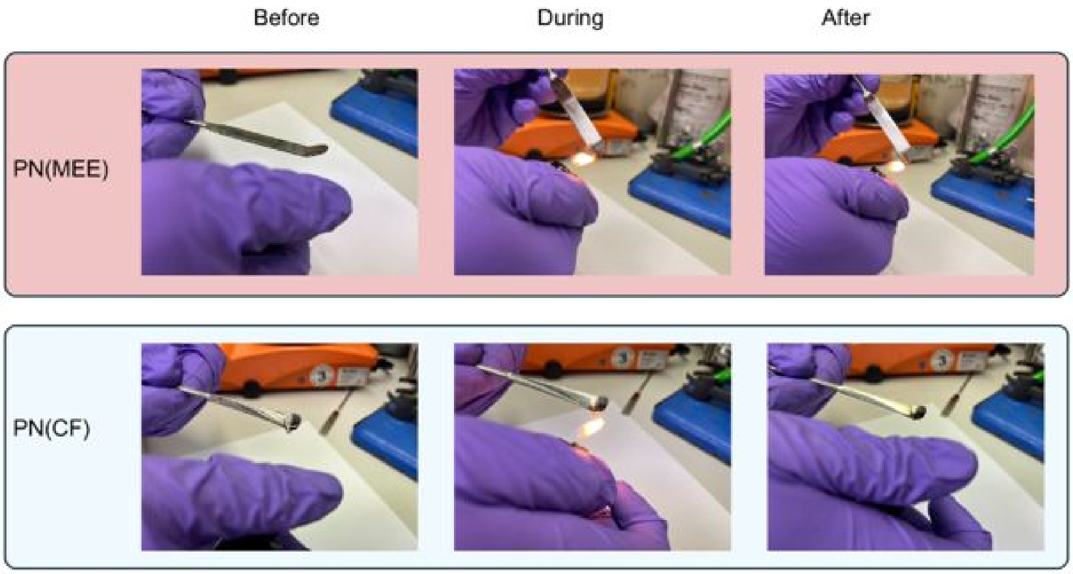

Figure S5: Images of a flame-retardant test of the two bulk polymers, PN(MEE) (top) and PN(CF) (bottom). Images show both polymers do not catch alight when exposed to the flame.

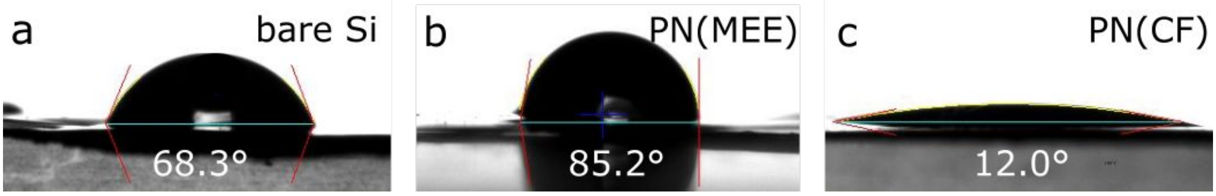

Figure S6: Water contact angles measured for the a) uncoated, b) PN(MEE)-coated and c) PN(CF)-coated silicon thin-film anodes. Reproduced with permission.[1] Copyright 2020 N.-J. H. Kneusels.

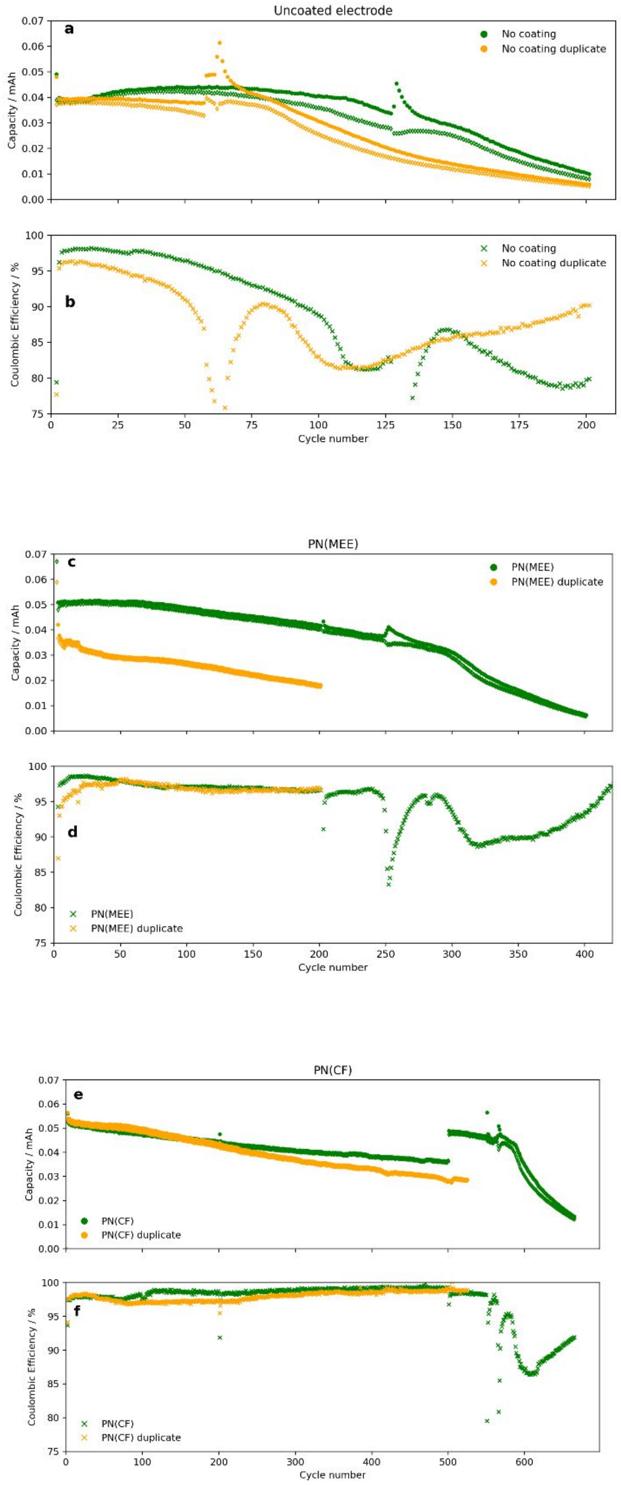

Figure S7: Electrochemical performance of the duplicates for a) uncoated electrode (the electrodes duplicate cut-off voltage was lowered to 45 mV after 125 cycles), b) the PN(MEE) coated electrodes and c) the PN(CF) coated electrodes.

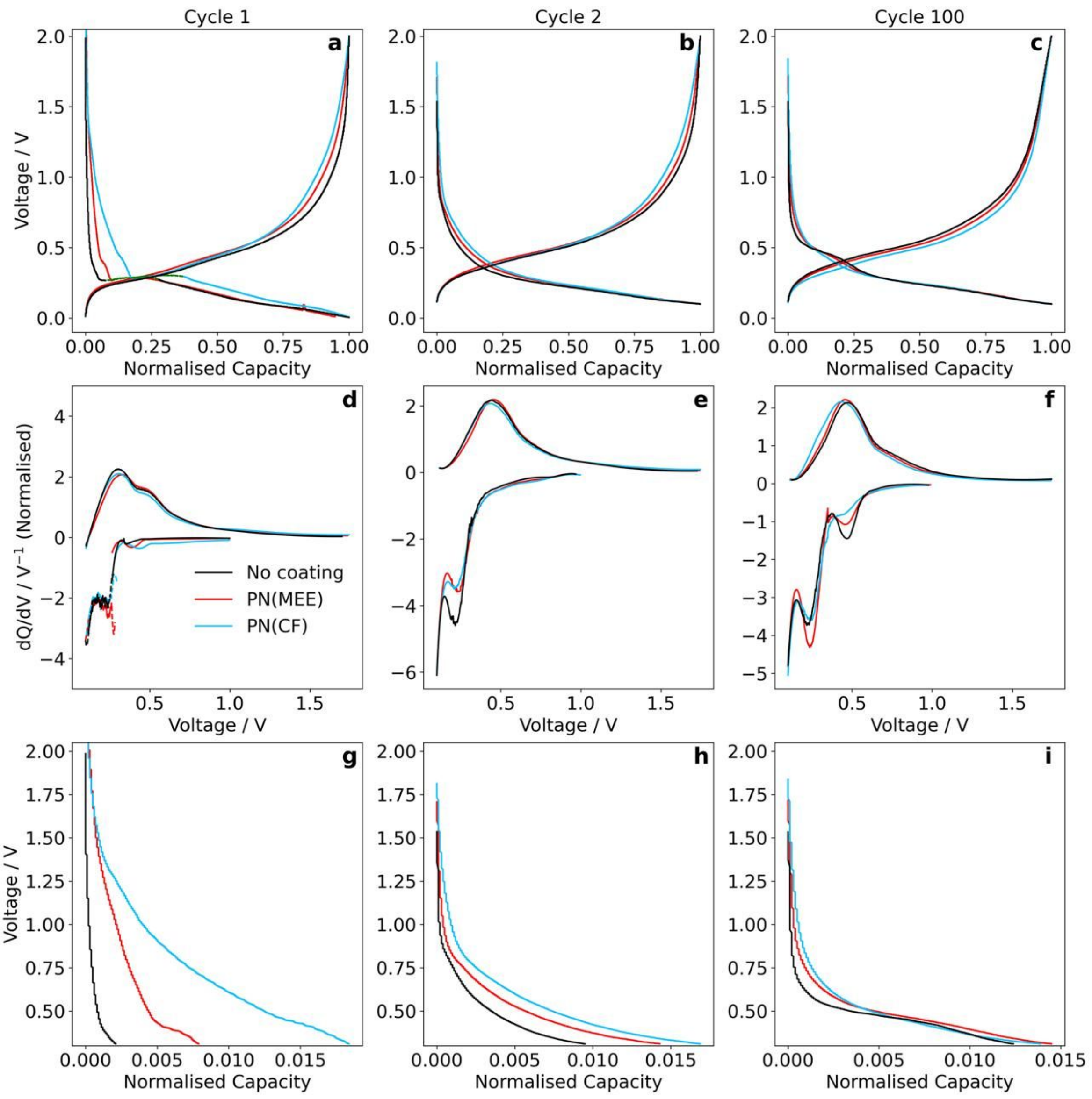

Figure S8: Normalised capacity plots for all three samples. a) 1st cycle, b) 2nd cycle, c) 100th cycle; d)-f) respective differential capacity plots; g)-i) high voltage region of the lithiation process of the respective plots.

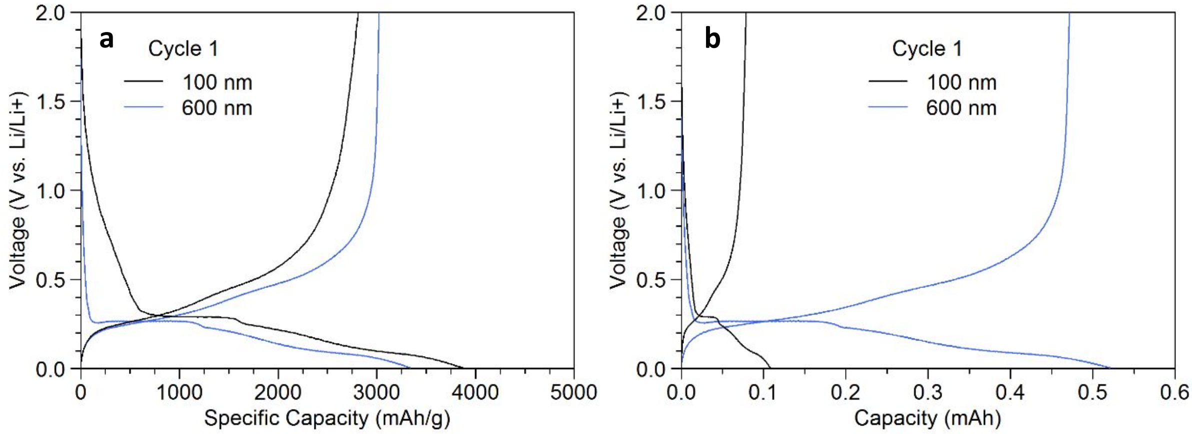

Figure S9: Voltage profiles for the first cycle of different thickness (100 nm and 600 nm) Si thin-film electrodes. a) Relative capacity plot, b) absolute capacity plot. Reproduced with permission.[1] Copyright 2020 N.-J. H. Kneusels.

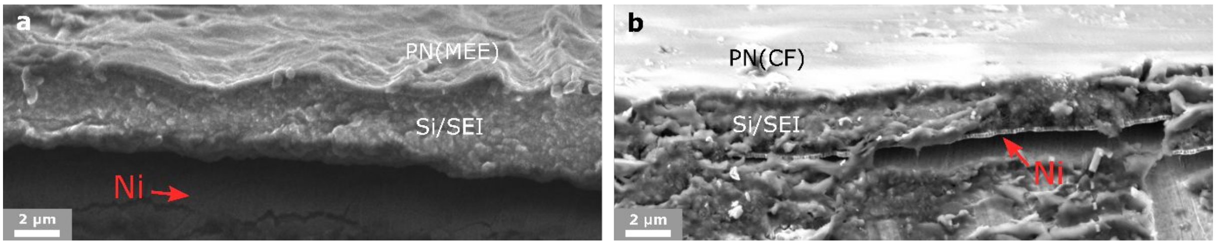

Figure S10: SEM micrographs of the blade-cut cross section of the 100 nm *a*-Si thin-film anodes on Ni-coated CR Cu coated with a) PN(MEE) after 500 cycles and b) PN(CF) after 525 cycles. Reproduced with permission.[1] Copyright 2020 N.-J. H. Kneusels.

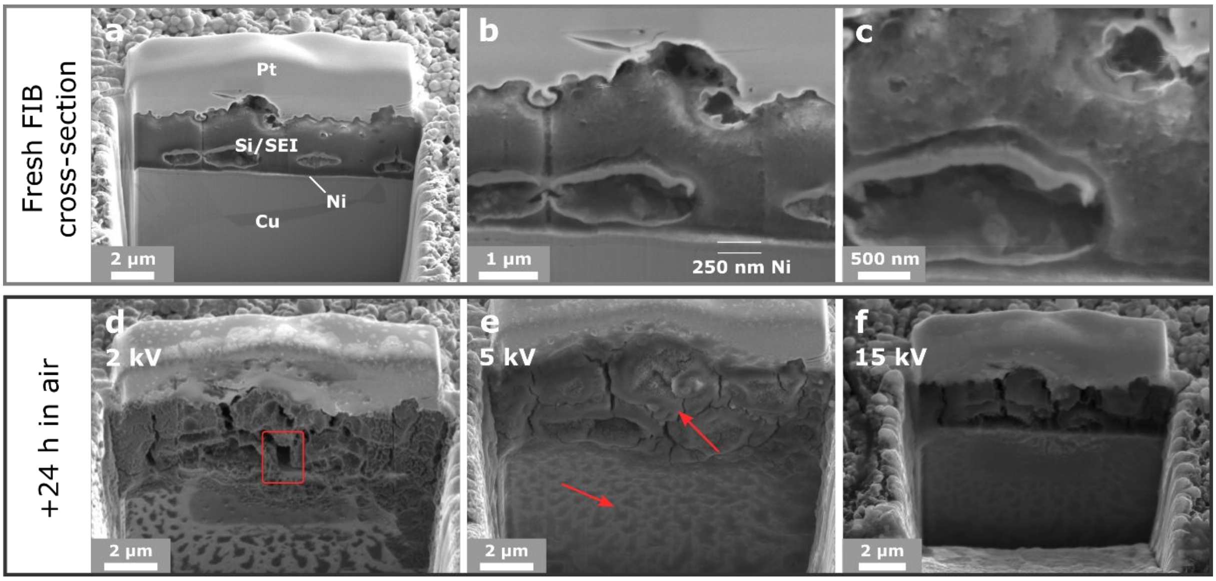

Figure S11: a-c) SEM micrographs (5 kV) of the FIB cross section of a 100 nm *a*-Si thin-film anode on Ni-coated CR Cu after 200 cycles. The 250 nm nickel layer is marked in b) as a vertical reference. d-f) Micrographs of the cross section under different acceleration voltages after exposure to air (2 kV, 5 kV, 15 kV; beam damage in d) and f) was inflicted by the 15 kV electron beam; order of acquisition: e - f - d; the highlighted area in d) clearly shows the beam damage from the long exposure during EDS. The arrows in e) show the bulging material and the dried electrolyte stains. The surface was coated with several nanometres of gold followed by a platinum bar (~2 µm) on the FIB milling site. Micrographs were recorded at an angle of 53°. Reproduced with permission.[1] Copyright 2020 N.-J. H. Kneusels.

<u>Reference</u>

[1] Kneusels, N.-J. H. Understanding and Preventing the Degradation of Silicon as a Lithium Ion Battery Anode. Ph.D. Thesis, University of Cambridge, Cambridge, U.K., 2020. https://doi.org/10.17863/CAM.49835.
